# Cancer stem cells as key drivers of tumour progression

**DOI:** 10.1186/s12929-018-0426-4

**Published:** 2018-03-06

**Authors:** Ain Zubaidah Ayob, Thamil Selvee Ramasamy

**Affiliations:** 1 Stem Cell Biology Laboratory, Department of Molecular Medicine, Faculty of Medicine, University of Malaya, 50603 Wilayah Persekutuan Kuala Lumpur, Malaysia; 2 Cell and Molecular Laboratory (CMBL), The Dean’s Office, Faculty of Medicine, University of Malaya, 50603 Wilayah Persekutuan Kuala Lumpur, Malaysia

**Keywords:** Cancer stem cells, Resistance, Stemness, Tumour microenvironment, Extracellular matrix, Hypoxia, Exosomes, Quiescence, Angiogenesis, Metastasis

## Abstract

**Background:**

Cancer stem cells (CSCs) are subpopulations of cancer cells sharing similar characteristics as normal stem or progenitor cells such as self-renewal ability and multi-lineage differentiation to drive tumour growth and heterogeneity. Throughout the cancer progression, CSC can further be induced from differentiated cancer cells via the adaptation and cross-talks with the tumour microenvironment as well as a response from therapeutic pressures, therefore contributes to their heterogeneous phenotypes. Challengingly, conventional cancer treatments target the bulk of the tumour and are unable to target CSCs due to their highly resistance nature, leading to metastasis and tumour recurrence.

**Main body:**

This review highlights the roles of CSCs in tumour initiation, progression and metastasis with a focus on the cellular and molecular regulators that influence their phenotypical changes and behaviours in the different stages of cancer progression. We delineate the cross-talks between CSCs with the tumour microenvironment that support their intrinsic properties including survival, stemness, quiescence and their cellular and molecular adaptation in response to therapeutic pressure. An insight into the distinct roles of CSCs in promoting angiogenesis and metastasis has been captured based on in vitro and in vivo evidences.

**Conclusion:**

Given dynamic cellular events along the cancer progression and contributions of resistance nature by CSCs, understanding their molecular and cellular regulatory mechanism in a heterogeneous nature, provides significant cornerstone for the development of CSC-specific therapeutics.

## Background

Despite the progress being made in the treatment for cancer, cancer remains one of the most common causes of death globally. Cancers are most likely curable when they are diagnosed at the earlier stage through conventional treatments such as surgery, chemotherapy and radiotherapy. However, many cancers are also diagnosed at a later stage, during which the cancer have become progressive and metastasize to other organ. Even if the cancer is diagnosed and treated at earlier stage, some residual cells still remain and following some time, may cause tumour recurrence and the cancer often becomes more aggressive which leads to metastasis. Growing evidence have implicated that these residual cells which could be found during any stage of cancer progression that are responsible for causing the therapeutic resistance, possesses stem-like properties/functions known as the cancer stem cells (CSCs). Hence, this population of cells represents the critical subset within the tumour mass in perpetuating the tumour, even after what seems to be effective therapy and leads to tumour aggression. In the recent decades, the CSC theory generates much attention and excitement, whereby scientist believed this theory will revolutionize our understanding of the cellular and molecular events during the cancer progression contributing to therapy resistance, recurrence and metastasis. The CSC theory of cancer progression presents tumour as a hierarchically organised tissue with CSC population at the top rank in the hierarchy, that then generate the more differentiated bulk of the tumour cells with lower or limited proliferative potentials [1, 2]. CSCs share similar properties with normal stem cells, including the ability to self-renew and differentiation that give rise to heterogeneous, differentiated cancer cells making up the bulk of the tumour. Due to this similarity, CSCs are commonly characterised by the expression of surface markers associated with stem cells, such as CD133, CD44, CD90, and side population cells (SP) by which they can be isolated an enriched in vitro and in vivo, although no single marker can be used to define the CSC populations [2]. Also, their tumorigenicity potential is characterised by their enhanced ability to repopulate the original tumour when transplanted into immunodeficient mice even at low clonal density. Additionally, sphere forming assay were also used as an in vitro assay for the identification and enrichment of CSCs whereby only fractions of cells from solid tumours such as brain, breast, colon et cetera forms neurospheres, mammospheres and colonospheres respectively [3].

Despite extensive studies, there have been on-going controversies on the origin of CSCs, whether they arise from normal stem cells or non-stem cells [2, 4]. Additionally, their true phenotypes and functions remain argumentative. However, if the hypothesis of CSC being resistant population of cells is accepted, it may be possible that these cells are either; i) quiescent, non-dividing cells hence conferring their insensitivity, or ii) proliferative CSCs, but insensitive to the chemotherapy due to activation of resistance mechanisms. While many studies demonstrated that de novo CSCs exist in the tumour mass, it has also been proposed that CSCs is dynamic cellular states, a mechanism whereby acquisition of stem-like traits is necessary for them to be resistant and promotes tumour progression [5, 6]. Nevertheless, the tumour microenvironment plays an integral part during the tumour progression and metastasis, therefore presumed to support the cellular fate of CSCs [7].

**Fig. 1:**
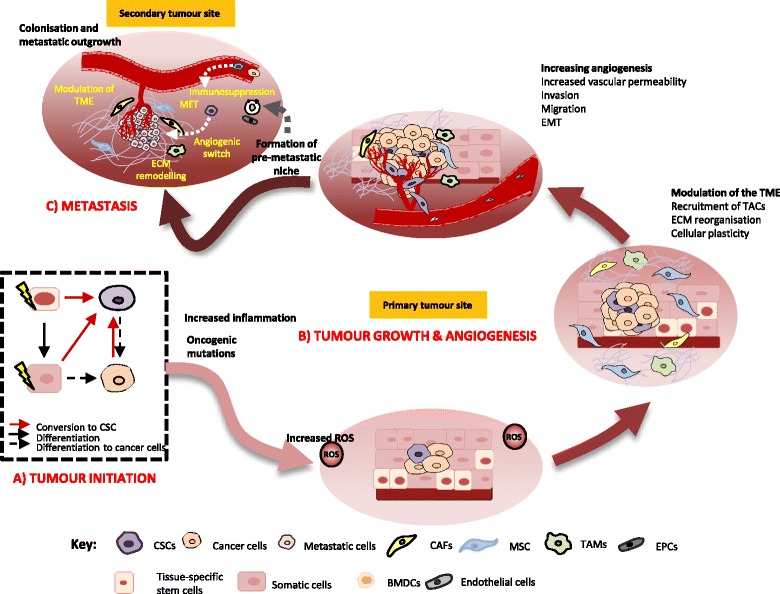
Summary of key cellular events during the progression of cancer from tumour initiation, tumour growth, angiogenesis and metastasis

Tumour progression involves complex cellular and molecular processes that are preceded by the initial genetic and epigenetic alteration causing the transformation to cancer cells. Conceptually, cancer can be divided into the following stages: initiation, promotion and progression and these stages are concomitant with complex and dynamic cellular events [8], as summarised in Fig. 1. Given the constant change in the structure and anatomy of the tumour as cancer progresses, increasing evidence shows that CSCs also changes in response to these dynamics, as an adaptive response for their survival [9]. This review therefore aims to dissect the behaviours and the roles of CSC; their regulation throughout the tumour progression, and the phenotypical outcomes resulting from these behaviours. Integral to the concept of tumour heterogeneity in cancer progression, CSCs therefore play an active role throughout the cancer progression as well as in therapy resistance by manipulating their intrinsic and extrinsic adaptation, favouring their growth and survival. Therefore, better understanding of CSCs behaviours, which differs according to their microenvironment corresponding to the different stages of cancer, is important in order to better devise more effective therapeutic strategy targeting these populations.

## CSC in tumour initiations

Carcinogenesis involves series of events, often initiated with cells losing their growth control due to accumulated mutations, leading to uncontrolled proliferation. This usually involves the alteration of gene such as oncogenes, tumour suppressor genes as well as those involved in DNA repair mechanisms. Subsequently, additional mutation results in the clonal selection with more aggressive phenotypes [10]. With no therapeutic intervention, the cancer becomes increasingly progressive, facilitated by the surrounding tumour microenvironment providing tumour growth supportive signals, the cancer no longer remains localised but begins searching for new soil for them to compensate increasing needs to survive, via a metastatic cascade. In this milieu, research has suggested that cancer cells are capable of influencing their surrounding tumour microenvironment to make it permissive for them to survive and evolve with more resistant and aggressive phenotypes as the cancer progresses. These events implicate why treatment outcomes are relatively poor and are more difficult to manage when patients are diagnosed at a higher stage of cancer. In another case, cancer may, more often, increase in aggression following therapeutic intervention such as chemotherapy, due to the presence of subpopulation of stem-like cells with resistance properties, subsequently capable of re-initiating the tumour, causing tumour relapse.

When the field of cancer research is ‘renewed’ with the CSC theory, whereby a subset of cells with stem cell properties are responsible for the perpetuation of cancer, a great amount of research has been done in identifying CSCs and understanding the mechanisms underlying their formation and thus, their roles in cancer initiation. Studies by Dick group in 1994 showed that leukemia-initiating stem cells present in the acute myelogenous leukemia (AML) patients could induce AML when transplanted into severe combined immunodeficient (SCID) mice [11]. The presence of stem cell in cancer were further supported in other studies in breast cancer [12], brain [13] and subsequently other types of cancers. Due to their having the self-renewal capacity and differentiation capacity in driving the tumour growth, CSCs are hypothesised to be originated from the normal stem cells/progenitor cells of the tumour tissues. Under normal physiological condition, normal stem cells usually reside in a quiescent state which is maintained by a specialised niche. Only upon receipt of a stimulating signal, the stem cells become activated to divide and proliferate. Any genetic mutation causing stem cells to become independent of growth signals, or to resist antigrowth signals, will cause the stem cells to undergo uncontrolled proliferation and possible tumorigenesis [4]. Direct evidence of the roles of CSC in cancer initiation is drawn from the many studies demonstrating the capacity of isolated CSCs, characterised by their positive expression of stem cells markers, to repopulate the parental tumour in immunodeficient mouse even at very low number whereas their negative, non-CSC counterpart does not exhibit similar tumorigenicity [14]. These studies supported one of the modes of CSC initiation, whereby transformed normal stem cells or progenitor cells could give rise to the formation of CSCs. Further insights into the mechanisms of CSC in cancer initiation have unravelled the roles of stemness transcription factors. For example, in a mouse model of skin carcinogenesis, Blanpain and colleagues demonstrate a key role for the transcription factor SOX2 in initiation and progression of melanoma. SOX2 is not expressed in normal skin, but it appears at an early stage in tumour formation. Tumour initiation can be prevented by deletion of the Sox2 gene. In addition, SOX2-expressing cells function as tumour propagating cells upon transplantation, while the removal of SOX2-postive cells from established tumours leads to regression. SOX2 appears to be able to contribute to both tumour initiation and progression by directly regulating genes involved in cancer functions such as stemness, proliferation, survival and invasion [15].

### Role of inflammation in cancer initiation

Central to all cancers is inflammation and that the cell processes involved in inflammation not only are responsible for initiation of the cancer, but also persist during its growth and play a central role throughout every phase of the cancer’s existence, including progression, invasion, angiogenesis, and metastasis [16, 17]. Carcinogenic events and conditions such as chemicals, obesity, hyperglycaemia, persistent infections, autoimmune diseases, and carcinogenic heavy metals, are known to promote inflammation [18] . Indeed, up to 20% of human cancers are accompanied with underlying virus infection; for example human papillomavirus (HPV-cervical carcinoma), herpes virus (lymphoma), hepatitis B and C (hepatocellular carcinoma), cytomegalovirus (glioblastoma), and *Helicobacter pylori* (gastric cancer) promote cancer development by inducing chronic inflammation [19]. Under inflammatory conditions, ROS and RNS can induce the formation DNA lesion products, including 8-oxo-7,8-dihydro-2′-deoxyguanosine (8-oxodG) and 8-nitroguanine respectively, which is considered to be mutagenic [20]. While it is known that multiple mutations accumulating over time are responsible for the malignant transformation of cells, there is significant evidence that increased generation of inflammation inducing factors such as reactive oxygen species (ROS), reactive nitrogen species (RNS), and lipid peroxidation products (LPPs) are the underlying damaging elements [17].

To link the possible roles of inflammation and CSC in the cancer initiation, evidence can be drawn from the changes in the microenvironment within the stem cell niche. It has been observed that the formation of CSCs is preceded by the transition of the stem cell niche into an area of high concentrations of ROS and RNS, LPPs, inflammatory cytokines and chemokines [17, 21]. Prolonged exposure of these stem cells’ DNA to assaults by ROS/RNS and LPPs can produce varying degrees of genetic mutations that over time is beyond repair, and these cumulatively may drive the conversion a stem cell into a cancer stem cell [20, 21]. Additionally, accumulating studies identifies co-localisation of CSC markers in inflammation-related cancers, as summarised in review by Ohnishi et al. [20], suggesting the possible roles of inflammation inducing CSCs. For example, expression of Oct3/4 and CD44v6, have been shown to be correlated in urinary bladder cancer induced by *Schistosomahaematobium* (SH) infection [22], whereas higher CD44v6 expression alone correlates with urinary bladder without the infection [22, 23]. In the same study, the authors also demonstrate that nuclear localisation of cyclooxygenase-2 (COX2) is associated with the upregulation of these stemness markers [23]. COX2 mediates the activation of prostaglandin E2 (PGE2) signalling, which is also involved in the inflammation-induced activation of normal stem cells or CSCs [24]. This SH-infection induced inflammation causing iNOS-independent DNA damage, promotes the expansion of mutant stem cells, via NF-κB activation leading to tumour development [25]. Similarly, cholangiocarcinoma tissues with underlying *Opithorcis viverrini* infection positively express CD133 and Oct3/4, suggesting stem cells are involved in the initiation of cancer via inflammatory inductions [26].

Taken together, transformation of the normal stem cells or progenitor cells may define a key event in the derivation of CSCs thus directly contributes to the initiation of cancer. The underlying inflammation and oxidative stress induction represent key event leading to the accumulation of mutational events acquiring the CSC phenotypes, however their mechanisms need to be further explored. Importantly, acquisition of these stem-like, CSC phenotypes also occurs in the more differentiated cancer cells as the cancer becomes more progressive predominantly through the interactions with the microenvironment, which will be discussed further in the next section.

## CSCs in tumour growth and angiogenesis

As the tumour develops, it becomes increasingly important for the cancer cells to sustain their growth and functions achieved through formation of tumour microenvironment by recruiting cellular components and modulating their extracellular matrix (ECM). Additionally, the tumour mass is increasingly hypoxic due to increase in tumour size, causing the formation of new vasculatures to facilitate diffusion of nutrients and oxygens to the cancer cells through angiogenesis process. Thus, the niche plays key roles in CSCs maintenance by regulating their stemness properties via activation of key signalling pathways involved in the self-renewal, angiogenesis and promotes the long-term survival of CSC. CSCs however, do not play the passive roles of becoming the receiving ends but they work together in modulating the niche in their favour predominantly through their interactions with the components in the niche. As a mechanism of adaptation, CSCs can interact with their micro-niche to modulate their survival, growth and metastatic regime/desire in a stressful therapeutic pressure, an unfavourable environment especially during and after cancer treatments. Importantly, cancer cells and CSCs “educate” the surrounding cells such as the stromal and immune cells by secreting signals that recruit, transform and alter the functions and activities of the surrounding cells which in turn facilitate growth and progression of tumour [27]. This interactions, occurring through both cell-cell or ECM-cell communication ensures the niches balance between self-renewal, differentiation and resistance properties of CSCs [28]. These interplay of the different factors in the tumour microenvironment, including therapeutic pressures can also promote the induction of CSCs from non-CSCs, and seems to be mediated by common signalling pathways predominantly the Notch, NF-κB, TGF-β, Wnt/β-catenin, and MAPK signalling pathways, to name a few [29–31]. The regulation of the CSCs characteristics through these pathways by the different microenvironmental factors can be distinguished by the different mediators/effectors in eliciting the CSCs’ self-renewal and tumour promoting functions [31]. So how do these interactions promote tumour progression and what roles does CSC play in these interactions? In the next section, we highlight the interplay between CSCs and the niche components, including the tumour-associated cells (TACs), ECM, hypoxia and therapeutic pressures that contribute to the CSC characteristics and subsequently tumour progressions.

### Interactions with the cellular components in the tumour microenvironment

The key cellular components in the tumour milieu include the cells of mesenchymal origin such as cancer-associated fibroblast (CAFs), mesenchymal stem cell and endothelial cells and as well as of hematopoietic origins such as the macrophages, T-cells and natural killer (NK) cells. The cancer cells, CSC and TACs establish cytokine network that supports the maintenance of CSCs as well as promoting the formation of new CSCs, thus facilitating tumour survival, propagation as well as recurrence [32].

**Fig. 2:**
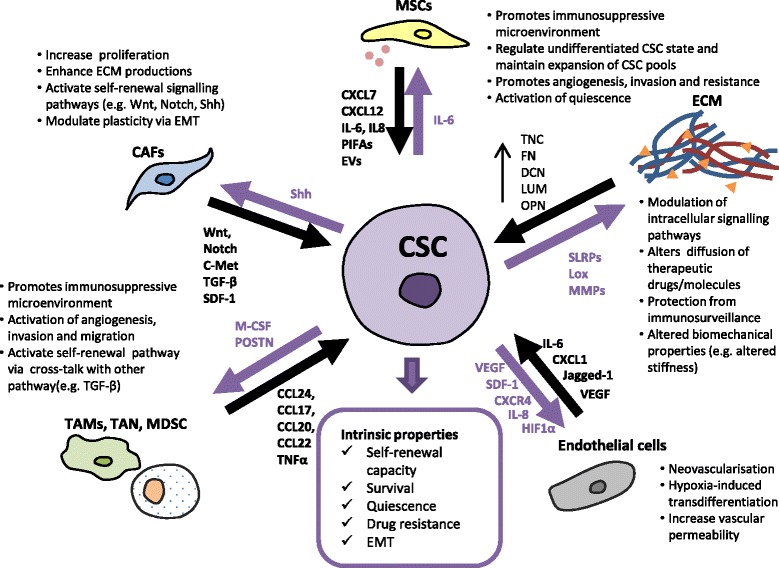
Cross-talk between CSCs and the components of TME. CSCs recruit and modulate the cellular components of the TME and reorganise the ECM in exchange for production of factors that drive tumour progression by promoting CSC intrinsic properties including stemness, survival, angiogenic, EMT and metastatic capacity

CAFs are resident cells within the tumour stromal which results from recruitment and transformation during tumour progression, supporting CSC function through autocrine and paracrine secretion of cytokines factors, as shown in Fig. 2. Cancer cells-derived secretory molecules, such as basic fibroblast growth factor (bFGF), transforming growth factor beta (TGF-β), platelet-derived growth factor (PDGF), and interleukin- (IL-) 6, transform surrounding fibroblast into CAFs [33–36], and in turn, CAFs promote tumour growth as well as sustain the stemness property of CSCs in a paracrine manner. Compared to normal fibroblast, CAFs are characterised by increased proliferation, enhanced production of ECM proteins and unique cytokines production [37]. In mammary tumour of murine models, Valenti et al. shows that CSC mediates the activation of CAFs via activation of hedgehog signalling [38]. In return, CAFs elicit their roles to maintain the self-renewal, expansion and plasticity of CSCs through paracrine signalling activation. For example, CAFs were shown to promote cancer stemness via the paracrine activation of Wnt/β-catenin and Notch pathways mediated by HGF in colon and liver cancer respectively [39, 40]. Studies have also demonstrated that CAFs are also involved in regulating the plasticity and maintain stemness properties of CSC by inducing epithelial mesenchymal transition (EMT), via paracrine secretion of TGF-β, stromal-derived factor 1 (SDF-1) [41, 42] and production of matrix metalloproteinase protein 9 (MMP9) [34].

Mesenchymal stem cell (MSC) is another key cellular player that cross-talk with CSCs to promote tumour progressions by enhancing proliferation, fostering angiogenesis, promoting metastasis and most notably are responsible for generating an immunosuppressive microenvironment. For example, CSCs in breast cancer produce cytokines such as IL-6 to attract the MSCs, which then produce CXCL7. This key cytokine, in turn, induces the production of many other cytokines by tumour cells to support the growth of CSCs while suppressing immunological cascade [43]. MSC also promotes stemness through NF-κB pathways via secretion of CXCL12, IL-6, and IL-8 [30]. MSC stimulates tumour progression by production of Gremlin1 to promote undifferentiated state [44]. MSC also causes aberrant regulation of microRNA associated with CSC maintenance and survival. For example, MSC promotes the CSC properties by upregulating the expression of miR-199a, subsequently causing deregulated expression of a network of microRNAs and suppressed Forkhead box protein P2 (FOXP2) [45]. Evidence has demonstrated the roles of MSC in drug resistance, mediated by the release of platinum induced fatty acids (PIFAs) responsible for their platinum drug resistance; activation of SDF-1α/CXCR4 signalling or by converting themselves into CSCs [46]. Cross talk between both CSC and MSC established through extracellular vesicles (EV) or exosomes secretion is implicated to facilitate tumour angiogenesis, invasion, drug resistance, as well as activation of dormant or quiescent cancer cells [47, 48]. MSC-derived EV (MSC-EVs) has been shown to elicit both tumour promoting and inhibitory effects, whereby it can enhance the tumour growth and metastasis, or they may promote apoptosis of cells and cause tumour regression [48–51]. For example, MSC-derived EV can induce the activation of ERK1/2 signalling pathway to promote growth of renal carcinoma, leading to the progression from the G0/G1 to S phase of the cell cycle [52]. Additionally, MSC-EVs were also shown to promote the metastatic phenotypes in MCF7 breast cancer cell line, which is achieved by activating signalling pathways including ERK1/2 [53] and Wnt/β-catenin pathways [54], suggesting the enrichments of CSC phenotypes.

The tumour microenvironment is also characterised by chronic inflammation that promotes the tumour proliferation and metastasis through immunosuppression and evasion from immune surveillance [55]. Cancer cells and CSCs promote an inflammatory niche by secretion of chemokines and cytokines to recruit tumour-associate macrophages (TAM), tumour associated neutrophils (TANs), and myeloid-derived suppressor cells (MDSCs). For example, IL-13, IL-34 and osteoactivin derived from cholangiocarcinoma CSCs sphere conditioned media could promote the activation of CD14+ macrophage with TAM-like features and exhibit in vivo tumour promoting effect [56]. In other instance, glioblastoma stem cells secrete periostin (POSTN) to recruit M2 macrophage-tumour supportive macrophage, and disruption of POSTN leads to the specific inhibition of the tumour-supportive M2 types of TAMs in xenografts [57]. Additionally, TAMs elicit their pro-tumour activity by activation of angiogenesis and promotes migration and invasion via induction of EMT. For examples, production of chemo-attractants by TAMs such as CCL24, CCL17, CCL20, CCL22 stimulate pro-angiogenic capacity, in response to macrophage colony stimulating factor (M-CSF) secreted by tumour cells [58, 59]. TAMs and CD4+ T cells secrete TNFα which upregulates NF-κB signalling pathways to induce EMT-associated transcription factors such as Slug, Snail and Twist, increasing the crosstalk with the TGF-β signalling pathway promoting self-renewal, migration and invasion of CSCs. Recently, exosomal transfer of microRNA have been implicated to modulate the activation and reprogramming of TAMs [60, 61] and in return, TAM-derived exosome transfer of miR-21 confers drug resistance in gastric cancer cells [62].

### Role of hypoxia in tumour progression

Hypoxia is one of the key features of solid tumour characterised by reduced oxygen levels (< 2%), resulting from high oxygen demand from proliferating cancer cells and low oxygen supply due to irregularities in tumour vascularization or distance from supporting blood vessels. Hypoxia-inducible factors (HIFs), including HIF1α and HIF2α represents the primary mediators for the cellular response to hypoxic condition by regulating diverse cellular processes during cancer progression including survival, proliferation, metabolism, EMT, angiogenesis and metastasis [63, 64] . High expression of HIFs have been correlated with poor prognosis in various tumour types [65, 66]. With respect to the roles of CSCs in cancer progression, the hypoxic conditions were also linked in regulating the CSCs biology including in the maintenance of self-renewal/stemness, EMT, quiescence and drug resistance properties [67]. Several reports using in vitro studies have indicated non-CSCs also acquire the stem-like characteristics through the expression of genes such as OCT4, SOX2 and NANOG, which is required for the maintenance of self-renewal in stem cells or the activation of the Notch signalling pathway that regulates cell self-renewal and differentiation [68–70]. Intriguingly, the hypoxia-associated CSC enrichment have been shown to be primarily dependant on the HIF2α pathway [71]. Other signalling pathways implicated in the regulation of stemness phenotypes by the HIFs included the TGF-β, Wnt/β-catenin, TNFα and NF-κB [72–74]. Activation of these signalling pathways is also implicated in the induction EMT via the transcriptional control of EMT-associated transcription factors such as SNAIL, TWIST, ZEB1, SLUG and TCF3 [75, 76]. Additionally, hypoxic condition also promotes high ROS in the tumour microenvironment, subsequently leads to the activation of stress signalling in CSC mediated by TGF-β and TNFα signalling pathway that maintain their undifferentiated state [77–79]. Under hypoxic condition, both the CSCs and stromal cells activate their HIF genes, which are the primary factors that drive angiogenesis via the induction of VEGF [55, 80]. Given that the maintenance CSCs are not only favoured but enriched under hypoxic condition, the development of therapeutics targeting the HIFs and the associated pathways represent an attractive approach to target these populations.

### Tumour angiogenesis

TACs collectively support the angiogenesis during the tumour progression and this process predominantly involves endothelial cells and pericytes [81]. Cross-talks between endothelial cells and CSC, established through their proximity with the blood vessel, predominantly support angiogenesis via secretion of pro-angiogenic factors, and promote the expansion and maintenance of CSC phenotypes and survival via juxtacrine signalling activation of self-renewal pathways [82–84]. For example, endothelial cells promote the expansion of CSCs via expression of soluble form of Jagged-1 and Shh ligands to activate the Notch and Sonic Hedgehog pathways respectively [85, 86]. Human oesophageal cancer endothelial cells can enhance migration, invasion and self-renewal properties of oesophageal carcinoma cell in vitro by a direct cell-cell interaction through enhance epiregulin expression [87]. Krishnamurthy et al. observed that IL-6 levels in tumour-associated endothelial cells define the tumorigenicity of CSC in head and neck squamous cell carcinomas, as evidenced by enhance sphere formation and promotes stemness phenotypes via phosphorylation of STAT3 pathway [88]. In lymph nodes associated metastasis, lymphatic endothelial cells promote angiogenesis and lymphangiogenesis via secretion of CXCL1, increasing vascular permeability in pre-metastatic organs as well as mediate immunosuppression by recruiting immature dendritic cells via CCL21 expression [89, 90]. The angiogenic activity of endothelial cells is also regulated by diverse microRNA that has been shown to have both pro and anti-angiogenic activities [91]. Endothelial cells are also shown to induce EMT which confers them stem cell properties and lower sensitivity towards anti-cancer drugs [92, 93]. In return for this interaction, CSC also preferentially upregulates pro-angiogenic factors such as VEGF, stromal derived factors 1 (SDF-1), interleukin 8 (IL-8) and CXCR4 to drive the angiogenesis process [94]. Emerging evidence demonstrated that CSC also actively involved in angiogenesis by transdifferentiating into functional endothelial cells, shown in breast CSC [95] and glioma stem cells [96–98]. Study by Soda et al. found that glioblastoma initiating cells transdifferentiate into endothelial cells induced by a hypoxia activation of HIF-1α, but interestingly is independent of VEGF expression [98]. In another study, Wang et al. and Ricci-Vittiani et al. demonstrated that endothelial cells share similar genetic alteration in glioblastoma stem-like cells, and that these CSC-like cells could be induced to transdifferentiate in a Notch-dependant manner [96, 97]. These studies altogether suggest the roles of CSCs in promoting angiogenesis by directing their fate into the endothelial lineages. Thus, the development of anti-angiogenic therapy may have to be re-strategized as we begin to unravel the lineage plasticity of CSCs capable of creating their own vascular system to maintain stemness and their tumorigenicity.

### Reorganisation of the extracellular matrix component

Other than the cellular component, the tumour microenvironment also comprises of non-cellular component that maintains the behaviour of the malignancy, which is the ECM. The ECM is a key component in the tumour microenvironments that mediates the cross talks between tumour cells and the microenvironment to promote malignant phenotypes. ECM components in the niche comprised of macromolecules such as collagens, glycoproteins and proteoglycans as well as integrins [99]. The ECM mediates extracellular cues from the microenvironment to maintain the stemness properties of CSCs, or direct their differentiation into heterogeneous tumour phenotypes, through the regulation of signalling pathways [99]. Cancer cells and CSCs design their own microenvironment by extensive overexpression of various matrix components, to support their growth and behaviours such as altering the diffusion of therapeutic drugs and other cytotoxic molecules [100]. For instance, deregulated expression of tenascin-C (TNC), which is one of the ECM proteins in stem cell niche, has been associated with their roles in cancer progression such as angiogenesis [101], invasion [102] and metastasis [103]. Increased TNC expression has been shown to correlate with poor prognosis and decreased survival in glioma patients [104], and was identified as potential biomarker for CSCs in glioblastoma [105]. Overexpression of TNC has been shown to play a role in driving cancer progression and drug resistance in melanoma enriched with stem-like SP cells, and knockdown of TNC decreased the SP cells fractions and sensitize them to doxorubicin treatment [106] Moreover, breast cancer cells supports their metastatic initiating ability by overexpressing TNC, by enhancing the regulator of stem cell signalling musashi homolog 1 (MSI1) and leucine-rich repeat-containing G protein-coupled receptor 5 (LGR5), suggesting this cancer cells derived TNC supports the metastatic-initiating breast cancer cells through enhancing the self-renewal pathways [103]. Additionally, Jachetti et al. demonstrated TNC protects prostate stem-like cells from immunosurveillance during dissemination via the suppression of T-cell receptor-dependent T-cell activation [107]. Similarly, Farace et al. demonstrated that the small leucine-rich proteoglycans (SLRPs) decorin (DCN) and lumican (LUM) is upregulated in CSCs enriched spheres derived from glioblastoma and neuroblastoma [108]. They also found that these increased in SLRPs is concomitant with acquisition of resistance to temolozomide drug, quiescent phenotypes and induce cellular heterogeneity by promoting dedifferentiation of cancer cells towards stem-like phenotypes, allowing their survival in ensuring the progression of cancer [108].

Indeed, ECM molecules and their associated receptors modulate the CSC behaviours not only through modulation of cell-cell signalling and their immunomodulatory roles, but the biomechanical properties of the ECM also determine how a cell senses and perceives external forces and thus provides major environmental signals that regulate cell behaviours. Alteration and remodelling of ECM structure is done by enzymes that digest the ECM, which are secreted within the tumour microenvironment. CAFs play key roles in altered activities of ECM remodelling enzymes and deregulated ECM metabolism through elevated expression of MMPs to mediate the degradation of ECM to facilitate cancer cells invasion [109, 110]. On the other hand, it has been shown that the ECM in the tumour stroma is typically stiffer compared to normal stroma, indicating the ECM biomechanical properties changes under different stroma condition and hence are constantly changing to drive the tumour progression [111, 112]. Under hypoxic condition, expansion of stem like cells is concomitant with 10-fold increase in LOX expression which is involved in generating the cross-linking molecules in the ECM, suggesting the role of CSC in promoting the stiffening of the tumour stroma facilitated by hypoxic microenvironment [59]. This suggests that the dynamics in the ECM deregulation promote the cancer progression, partly by expansion of CSCs, therefore may be a likely target for development of CSC-based therapeutics.

### Drug-induced resistance

Tumour relapse remains the biggest challenge for the management of cancer patient as often this is accompanied with more aggressive cancers and metastasis. Chemotherapy remains one of the main therapeutic modalities; however, the effectiveness is limited by chemoresistance. Resistance can be classified into two categories namely the intrinsic *(*de novo*)* and acquired resistance [113].

One of the most important features pertaining to the CSCs properties is their ability to resist to conventional therapies. The postulation that some cells remain following therapeutic pressures suggest the de novo population of cells that is unresponsive to the treatment, whereby the residual cells are enriched with the stem-like properties causing the tumour relapses and metastasis, suggesting the role of CSC [29, 114, 115]. Molecular mechanisms of chemoresistance in cancer have been well described which includes increased drug efflux rate, altered drug metabolism their ability to resist DNA damage due to enhanced repair capacity. Epigenetic changes as well as the influence from tumour microenvironments providing survival signals also contribute to chemoresistance [116, 117]. The presence of CSCs has been described as the mechanism through which chemoresistance is implicated, however many parallels have also been found to be associated with the aforementioned mechanisms. For example, CSCs are intrinsically resistant to conventional chemotherapies due to high expression of ATP-binding cassette (ABC) transporter proteins [118–120], enhanced aldehyde dehydrogenase (ALDH) activity [121], increased expression of anti-apoptotic proteins such as Bcl-2 and Bcl-X_L_ [122], enhanced DNA damage repair by activation DNA damage checkpoints such as CHK1 and CHK2 [123] and activation of key pro-survival signalling molecules [124, 125]. These establish that the acquisition of stemness traits is strongly associated with resistant or unresponsive properties in cancer.

Particularly, the quiescence nature of CSCs is of the interest of much research as this implies that treatment failure is inevitable as long these quiescence cells are present in the tumour bulk. Quiescence properties are often associated with stemness, as the normal stem cells reside in the G0/G1 stage of the cell cycle progression is a protective mechanism from cytotoxic stress [126]. In normal stem cell, cellular quiescence is an actively maintained and regulated state of the cells through a controlled signaling pathways and molecular regulators [126]. These include tumour suppressor p53 and retinoblastoma protein (RB), cyclin-dependent protein kinase inhibitors (p21, p27, and p57), Notch-related pathways, and a number of miRNAs (such as miR-126, miR-31 and miR-489) [126]. Using retention of DNA label or lipophilic dyes, the presence of quiescence CSCs have been demonstrated in many in vitro and in vivo studies of breast [127], liver [128], melanoma [128], glioblastoma [129, 130] and ovarian cancer [131, 132]. Activation of p21 via BMP signaling is one mechanism for resistance to chemotherapy- and radiation-induced cell damage [133]. However, in order to develop a therapy that targets these quiescence CSCs, one have to ask these questions: 1) Does these quiescence cells represents pre-existing population in the tumour? 2) Or is quiescence a result of cellular response of pre-existing, non-quiescence CSC in response to the therapy; or 3) does the cancer treatment selectively induce non- CSC to become quiescence, hence becoming unresponsive to the treatment? This quiescence-associated resistance also implies the temporal nature of CSC enrichment, in addition to different molecular mechanisms and therefore requiring a careful strategy to develop the therapy.

Regardless, these resistance and stemness-induced drug response often result in more aggressive phenotypes, which subsequently lead to tumour relapse and metastasis. The acquisition of these aggressive phenotypes is prominently implicated by the acquisition of mesenchymal phenotypes through the epithelial mesenchymal transition (EMT) [134–136]. Activation of EMT through the transcription factors mainly Snail, Twist, Slug, and Zeb is mediated through various signaling pathways, however common signaling pathway that have been implicated is through the TGFβ/Smad, PI3K/AKT, ERK1/2, inflammatory associated NF-κB [137, 138] pathway as well as the self-renewal pathways; WNT/β-catenin and Notch pathway [139]. Activation of EMT is also implicated as a molecular program in the initiation of metastasis, which causes the cancer cells to gain invasive and migratory potentials. In a report by Lee et al., treatment with chemotherapy drugs induced adaptive drug resistance and increased CXCR4^high^ cells with metastatic potential in ovarian cancer cell lines, whereas removal of drugs causes the cells to revert the state transition. Additionally, CXCR4^high^ cell exhibits dormancy in drug resistance and mesenchymal-like invasion, migration, colonization, and tumour formation properties [140].

Intriguingly, accumulating evidence supports the idea that non-CSCs can be induced into a transient, stem-like state enabling them to become drug-tolerant, involving reversible phenotypic switching through which EMT is also implicated. For instance, non-CSC cells in hepatocellular carcinoma exposed to carboplatin treatment acquired stem-like properties characterized by increased pluripotency marker expression (Sox2 and Oct3/4) and tumoursphere formation capacity [141]. Using both bladder and breast cancer cell lines model, He et al. demonstrated that cancer cells acquire a drug resistant, highly tumorigenic, cancer stem-like phenotype through modulation of the PI3K/Akt/β-catenin/CBP pathway [142]. Goldman et al. demonstrated that exposure of human breast cancer explants and cancer cell lines to high dosage of taxanes induce phenotypic change towards CD44^hi^CD24^hi^ [143]. This state transition conferring the cells with chemotherapy-tolerant properties is dependent on Src kinase signaling pathway and suppression of apoptosis [143]. In another study, drug-tolerant phenotypes are acquired transiently via engagement of IGF-1 receptor signaling and a high expression of epigenetic modifiers such as histone deacetylases (HDAC), and inhibition of either HDAC or IGFR disrupted CSC plasticity and re-sensitize the cells to drug treatment [144]. Taken together, the ability of cancer cells/CSC to transition between distinct cell states indicates the dynamics and the heterogeneity of an adaptive mechanism in response to cancer therapies.

Deregulation of cellular energetics have been implicated as one of the emerging hallmarks of cancer, as cancer cells intrinsically differed in their energy metabolism compared to the normal cells [145]. Until the recent years, many studies are emerging to elucidate the metabolic landscape in CSC, as metabolic reprogramming/switch may have a vital role in the acquisition of stemness, therefore conferring their resistance mechanisms. While CSCs also utilise aerobic glycolysis similar to cancer cells, studies have shown CSC switch their energy metabolism to an enhanced glycolytic via upregulation of GLUT1 transporters and resetting of their mitochondrial activity [146–149], similar to embryonic stem cells. This increase in glycolytic activity have been shown to increase the SP fractions with enhanced ABCG2 expression, mediated by ATP-mediated suppression of AMPK and activation of the Akt pathway [148]. Additionally, the ability to shift their metabolism is also profound even under different oxygen condition. Hypoxic or low oxygen condition have been implicated in inducing EMT, acquisition of stemness, resistance, promotes tumour aggression and induce metabolic switch [67]. Hypoxia-induced EMT enriching CSCs population is associated with increased expression of glycolytic genes, reduction in oxygen expenditures, reduced mitochondrial mass and membrane potential, and decreased production of ROS, with majority of the population residing in G1 phase of the cell cycle [149]. These show that metabolic reprogramming also plays important roles in regulating cellular fate in CSCs, which then contributes to their resistance properties. Metabolic reprogramming may represent an important intrinsic CSC regulation and glycolytic inhibition may be an attractive strategy to target the CSCs.

**Fig. 3:**
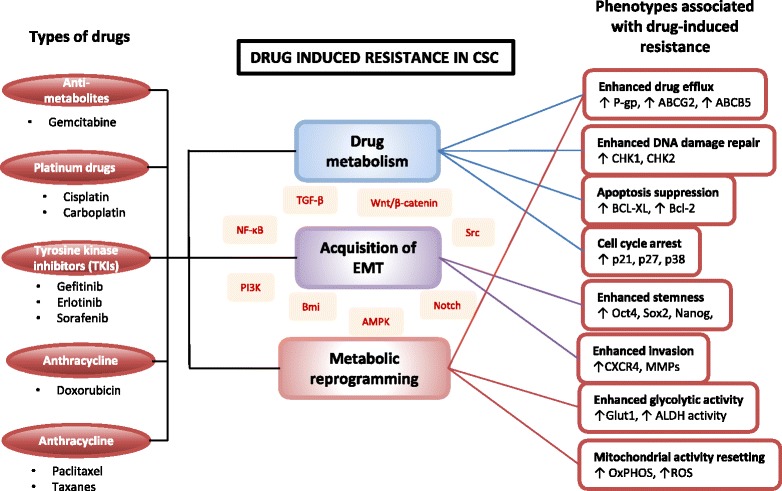
Summary of key CSC-associated phenotypes via modulation of drug metabolism, acquisition of EMT and metabolic reprogramming involving signalling pathways regulation in response to different types of chemotherapeutics, conferring CSC the resistance properties

Taken together, resistance to therapeutic pressures such as chemotherapy present one of the main factors that contribute to tumour aggression by enriching the cells with stem-like characteristics, which is modulated via various adaptive mechanisms, summarized in Fig. 3.

## CSC in metastasis

Metastasis is the development of secondary tumour growth at a distant organ or tissues from the primary tumour site, and is responsible for more than 90% cause of cancer related deaths [150]. Due to their resistant nature, CSCs are therefore inherently capable of metastasizing; this population known as metastatic CSC [150–152]. This section will provide insight into the roles of CSC in metastasis with a focus on the cellular and molecular mechanisms underlying the dissemination of metastatic CSCs.

Metastasis involves complex series of cellular and molecular events, characterised by local invasion, followed by intravasation into the blood and lymphatic system and then localisation and adaptation of disseminated cancer cells in the new metastatic site. However, what are the triggers for cancer cells to metastasise, and why do they go where they go? In 1989, the ‘seed and soil’ hypothesis for metastasis by Steven Paget’s established pivotal framework whereby both the intrinsic properties of the cancer cells i.e. the ‘seed’ and receptive microenvironment i.e. the ‘soil’ is required for the successful engraftment at the distant tissue and form metastases [153, 154]. It has been demonstrated that metastatic capacity may be a pre-determined, intrinsic traits in the cancer cells during the earlier phase of cancer rendering them with survival advantage [155]. Molecular signatures of primary tumour predicting poor prognosis due to metastasis have been defined in many tumour types, suggesting metastatic gene signatures is a shared property among cancer cells in the primary tumours [156–159]. Indeed, stem-like gene expression signatures in primary tumour have been shown to correlate with metastatic and survival outcomes, suggesting that these metastatic cells may be of CSC origin. Riester et al. analysed mRNA expression data of histologically diverse cancer samples in comparison to gene expression in stem cell samples including human embryonic stem cells, human mesenchymal stem cells and CD34+ hematopoietic stem cells. The authors found that poor overall survival is correlated with gene expression signatures with the most similar expression to that of stem cells [160]. While it remains to be determined the extent of the overlap between these tissues specific metastatic program to the expression profile of CSCs, evidence have shown that metastatic cancer possess distinct stem-like gene expression signatures [161–163]. For instance, recent study by Lawson et al. found that early stage metastatic breast cancer cells possess distinct signatures, associated with increased expression stemness, EMT, pro-survival and dormancy signatures. Contrastingly, late stage metastatic cells exhibit genes signature more closely associated with that of primary tumour, with increase expression of differentiation markers and less stem-like [163]. In addition, transcriptional profiling shows that late stage metastatic prostate cancer shares a common signatures with prostate basal stem cells and is associated with invasiveness [164]. Taken together, these studies support the notion that metastasis is closely associated with the stem-like properties, suggesting important roles CSCs play in the metastasis process.

**Fig. 4:**
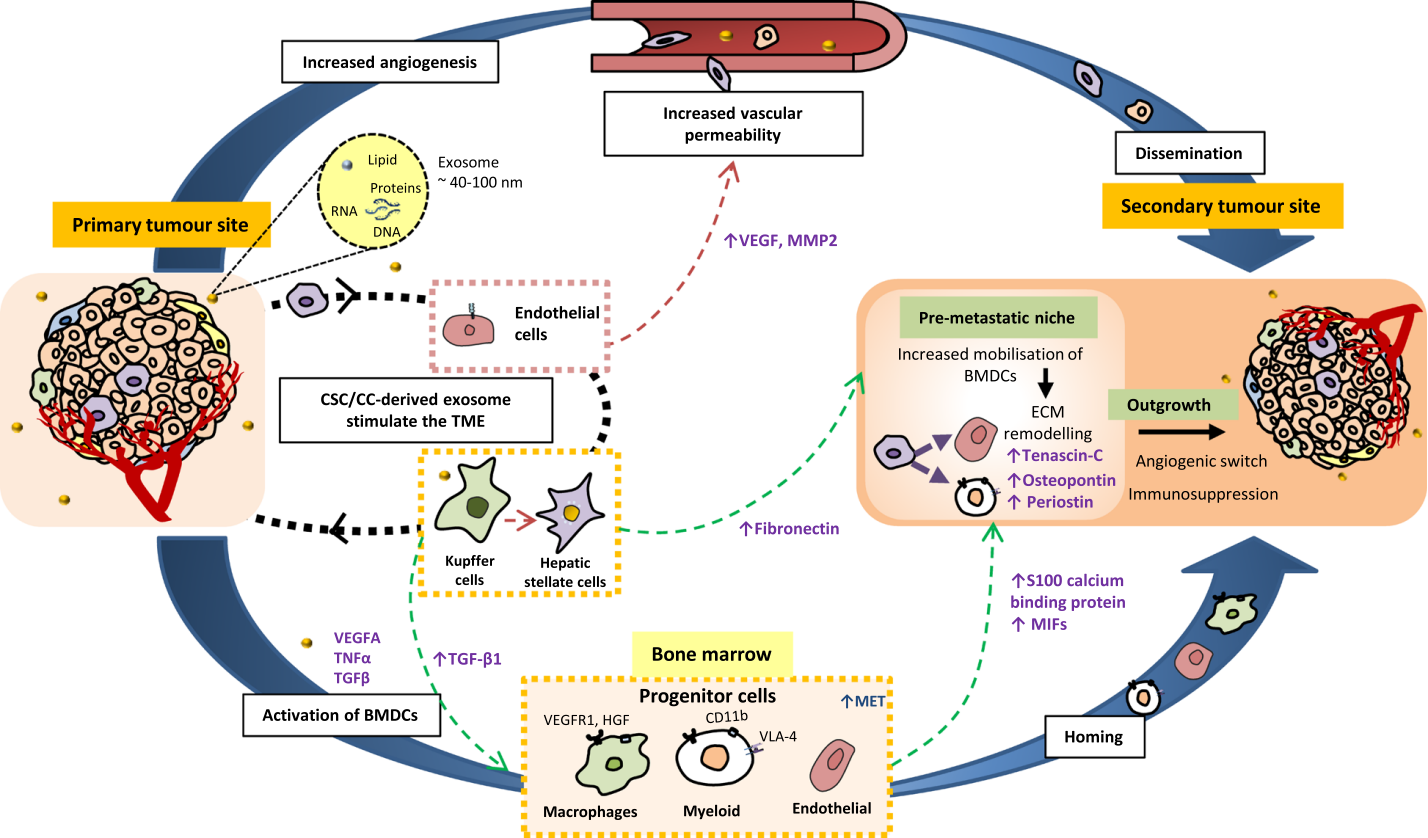
Cross talks between CSCs and the cells in the niche (e.g. endothelial cells, hepatic stellate cells, Kupffer’s cells) and bone marrow derived progenitor cells, BMDCs (myeloid progenitor cells, macrophage progenitor cells, endothelial progenitor cells) mediated by exosomal transfer of signalling molecules to initiate tumour metastasis by establishing the pre-metastatic niche. CSC further promotes the colonisation and metastatic outgrowth by modulation of dormancy, angiogenic switch and immunosurveillance

It has been demonstrated that CSCs play a role in the tumour microenvironment to orchestrate the metastasis cascade, via interactions with the cellular components of the tumour microenvironment to establish the new metastatic sites, termed the pre-metastatic niche for their arrival through a distinct cellular and molecular mechanisms [165, 166]. Subsequently, similar to the primary tumour, the microenvironment in the metastatic sites promotes the dissemination of malignant cells by creating a growth supportive niche and promoting angiogenesis to support the growth of the secondary tumour. In the next sections, we highlight the possible roles of CSCs in the context of pre-metastatic initiation and the metastatic outgrowth, as summarised in Fig. 4.

### Establishment of pre-metastasis niche

In the event of pre-metastatic niche establishment, cancer cells in the distant primary tumour sites hunt out new sites by secretion of factors and educating local cells including bone marrow derived hematopoietic progenitor cells (BMDCs), myeloid cells and endothelial cells, providing conducive foundation for their future seeding. It is known that VEGFR-1 expressing BMDCs are the key cellular components that are mobilized to site in the initiation of the pre-metastatic niche [167]. These cells express VLA-4(α4β1), which are recruited and mobilized by tumour-derived secreted factors such as placental growth factors (PlGF) and VEGF-A to activate resident fibroblast in the metastasis niche to prime the metastatic site in lung rich with fibronectins [167]. In addition, inflammatory cells such as CD113b expressing myeloid cells and macrophages were also recruited leading to the formation pre-metastatic niche through increased expression of S100 calcium binding proteins following induction by TNFα, TGFβ and VEGFA secreted by primary tumour cells [168, 169].

Exosomes are a class of tumour derived molecules of endocytic origin which are involved in the establishment of pre-metastatic niche Exosome are small, extracellular vesicles that carry diverse molecules, including proteins, lipids, RNA (mRNA, microRNA and other RNA molecules), as well as DNA molecules (dsDNA, ssDNA and mtDNA) [170]. Cancer cell-derived exosomes have been shown to have multiple roles in the events of metastasis, with a key role in the pre-metastatic niche formation through vascular remodelling and modulation of cellular behaviours in the pre-metastatic site (Fig. 4) [171]. For example, metastatic MDA-MB-231 breast cancer cells secrete exosomes enriched for miR-105 down-regulate tight junction zonula occludens 1 (ZO-1) protein expression, disrupting endothelial cell barrier and leads to increase vascular permeability, thus facilitate invasion and migration through intravasation [172]. Schilacci et al. demonstrate that exosomes derived from metastatic SW620 colon cancer cell line also enhance vascular permeability of surrounding endothelial cells via Rho/Rock pathway, in addition to promoting tumour progression by inducing phenotypic switch in the less aggressive tumour cells to confer metastatic behaviours [173]. In view of the roles of CSCs in exosomes mediated metastasis, it was shown that exosomes released from CD105+ renal CSCs activate angiogenesis and promote lung metastasis in vivo via the uptake and parallel up-regulation of VEGF and MMP-2 in lung endothelial cells [174]. Exosomes derived from highly metastatic melanomas were shown to increase the metastatic capacity by ‘educating’ marrow hematopoietic progenitors to express the receptor tyrosine kinase MET, which were then activated by hepatocyte growth factor (HGF) rendering BMDCs with higher migratory potentials and capable of establishing pre-metastatic niches [175]. Similarly, exosomes derived from pancreatic ductal adenocarcinomas are highly expressed in migratory inhibitory factors (MIFs) induces fibrosis and subsequently liver metastasis through cross-talks with Kupffer cells and hepatic stellate cells [176]. While there are increasing studies on the significance of exosomes-mediated cellular communication between CSCs and the tumour microenvironment promotes cancer progression, still little evidence is available on roles of CSC-derived exosomes implicated in regulation of metastasis.

### Colonisation and expansion of metastatic growth

During colonisation of distant organ, cancer cells in the primary tumour site invade the surrounding stroma and enter the vasculature (i.e. intravasation) [177]. The capacity for invasion and migration of cancer cells at the primary tumour site during the intravasation is facilitated by the EMT program [150]. In response to growth factor such as TGF-β1, activation of EMT allows the circulating tumour cells (CTCs) to translocate to distant site during metastasis, whereas mesenchymal-epithelial transition (MET) activation after extravasation may facilitate colonisation of the disseminated tumour cells (DTC) [178]. Increasing evidence in various cancer models suggests that a subpopulation of CTCs bears CSC phenotypes which intravasate and migrate together, suggesting the roles of CSCs during the initial metastasis process [179–183]. Indeed, the acquisition of the EMT phenotypes is also an important feature of CSCs which endow them with inherent metastatic potential [150]. Following the intravasation, CTCs transported via the blood stream to distant sites are arrested in the capillary bed, and subsequently extravasate through the microvascular walls to the parenchyma of the distant organ in which they may survive, proliferate and thereby establish metastatic colonies [184]. The successful sowing the newly DTC in the new ‘soil’ is influenced largely by the extrinsic factors i.e. of the microenvironment in addition to the cell-intrinsic factors, forming a metastatic niche that allows the survival and new tumour growth. The formation of metastatic niche constitutes adaptations and interactions of the seeding cells with the various niche associated cells, growth factors, soluble factors, inflammatory milieu, cytokine, enzymes, and ECM to facilitate the colonisation and metastatic outgrowth [165].

The adaptation period following dissemination is usually preceded by a period of dormancy in the DTC, which can last up to several decades [185]. Previous studies suggest that exit from the dormancy requires evasion from the immune surveillance mechanism that contributes to limit the outgrowth of micro-metastases and angiogenic switch to form micro-metastases, though the exact mechanism of DTC activation remains unclear [186–188]. Striking overlap exist between the behaviour of cancer cells in dormant state and behaviours of CSCs in tumour especially in the context of tumour outgrowth following metastasis dissemination, suggesting CSCs could be a subset of the dormant DTC [189, 190]. The regulation of dormant state is governed by a combination of intracellular and extracellular signals within the tumour microenvironment involving regulation of quiescence, alteration in angiogenic response and modulation of immune surveillance [189]. In the context of regulation of quiescence, equilibrium between the activation of p38/MAPK and ERK/MAPK is the key signalling determinant [191]. Additionally TGF-β and BMP signalling pathways, that regulate the maintenance of undifferentiated state in CSCs, also contribute to the maintenance of dormant state in tumour [192, 193]. For example, BMP7 secreted by bone stromal cells induced dormancy in prostate cancer stem-like cells [194]. BMP7 is also reported to induce CSC dormancy by activating p38/MAPK, p21, and N-myc downstream-regulated gene 1 (NDRG1) in a BMP receptor 2 (BMPR2)-dependent manner [191]. Hedgehog signalling pathways that govern the self-renewal properties sustain the CSC quiescence and stemness via upregulation of Bmi protein [195, 196]. Altogether, cancer dormancy that precedes the colonisation and metastatic outgrowth involves CSCs and development of therapeutic that manipulate this biology may be critical in halting the CSC-driven tumour recurrence. However, further studies are still needed to precisely elucidate the regulations of CSC dormancy in various cancer models, including their biology in different stages of cancer progression.

Angiogenesis induction represents a fundamental event underlying the switch from tumour dormancy to progressive cancer outgrowth. Bone marrow-derived endothelial progenitors cells (EPC) have been identified to be a critical cellular component that is recruited to mediate the angiogenic switch from micro- to macrometastasis and thus promotes metastatic outgrowth. [197]. The inhibitor of differentiation transcription factor, Id-1, which is a pro-angiogenic factors in primary tumour [198] is critical for the mobilization and recruitment of EPCs to micro-metastases. Stankic et al. shows that Id-1 also promotes metastasis by inducing mesenchymal to epithelial transition (MET) through antagonism with transcription factor Twist in lung metastasis, and overexpression of Id-1 induced by TGF- β generates breast cancer cells with CSC-like properties [199]. In addition to EPCs, other cell types such as TAMs also stimulate angiogenesis by expression of VEGF and angiopoietins, promote recruitment of other inflammatory cells, and secrete proteases to facilitate matrix remodelling [200]. Subsequently, progression of the metastasis outgrowth is potentiated by the cellular and molecular component of the metastatic niche. For example, in breast cancer metastasis to the bone, osteoblasts secrete the inflammatory cytokine interleukin 6 (IL6) induced by Notch activation contributes to metastatic outgrowth. Metastatic breast cancer cells in the bone benefit from CXCL12 and IGF1 which, through PI3K signalling, promote survival in metastatic cancer cells in a Src dependent manner [201]. In addition, metastatic outgrowth in breast cancer and melanoma were also promoted by various microRNAs by inducing recruitment of endothelial cells and angiogenesis [202, 203]. Oskarsson et al. show that breast cancer cell derived-TNC promotes the survival and outgrowth of pulmonary micro-metastases. TNC also enhances stem cell associated signalling Wnt and Notch, implicating CSCs may modulate the metastatic niche through TNC expressions [103]. Osteopontin (OPN), a glycoprotein that negatively regulates the pool size of HSCs in bone marrow, is also critical for breast cancer bone metastasis [204]. The expression levels of OPN in tumour microenvironment are regulated by CSCs, and, in turn, OPN modulate CSC phenotype via binding with CD44+ cells in promoting tumour progression and metastasis [205]. Additionally, CSC may secrete OPN to recruit bone-marrow derived cells to hijack the niches for normal stem cells or recruit new components to form a permissive niche including immune surveillance. Periostin (POSTN), another matricellular proteins are secreted by stromal cells to prime the lung stroma for CSC-supportive niche in response to TGF-β3 secretion by tumour cells [206]. POSTN recruits Wnt ligands (Wnt1 and Wnt3a), augmenting Wnt signalling in CSCs, which promotes CSC self-renewal and metastatic formation [206]. Taken together, these studies suggest CSCs promotes progression of metastasis growth by interacting with niche components to form permissive niche thus supporting their self-renewal.

## Future perspective: Challenges and opportunities

Tumour progression involves a dynamic changes and complex interactions within the tumour microenvironment that contributes to the maintenance of CSC phenotypes including resistance properties. CSC-based therapeutic are under the area of intensive research by targeting the different mechanisms that sustain the stemness and resistance nature of CSCs, and with a number of these drugs entering the early phase of clinical trials [207] These mechanisms includes (i) targeting cell surface markers of CSCs, (ii) targeting CSC signalling pathways (iii) microRNA-based therapeutics, (iv) targeting the components of tumour microenvironment, (v) immunotherapy and (vi) targeting CSC metabolisms. Developments of therapeutics that specifically target CSCs remain a crucial endeavour for successful eradication of cancer and not without great challenges, owing to their heterogeneity and cellular plasticity contributed by the different factors in the microenvironment. While CSC-specific targeting represents an attractive strategy as it may totally abolish cancer from recurrence, further studies are warranted in investigating mechanisms involved in cancer resistance to therapy and to determine whether the cells responsible for cancer relapse are similar to CSCs that initially perpetuate the tumour or as a results of resistance acquisition. As CSCs collaborate with the tumour microenvironment to favour their survival and resistance to chemotherapy, these interactions are not only important to understand treatment outcome, but could also provide useful targets for therapy.

Nevertheless, as discussed in this review, it is critical to evaluate whether this resistance is the results of discrete entities of CSCs or non-CSCs that transitioned to a more stem-like states to escape therapies which involve multiple mechanisms contributing to more tumour heterogeneity. Thus, as we begin to unravel the complexity of the tumour progression driven as the function of CSCs as well as their interaction with the tumour microenvironment, it presents a critical tool for pre-clinical studies and the importance of using the right models that recapitulate the in vivo cancer progression. On the other hand, CSCs heterogeneity may be an opportunity in the area of personalized medicine, therefore the development of novel assays to predict human tumour response to therapy will be helpful to choose the most appropriate treatment, increasing our chance to treat cancer more successfully.

## Conclusion

In this review we have summarised CSCs as a critical drivers of tumour progression, highlighting their behaviours and roles in the different stages of cancer which include tumour initiation, promotion and metastasis. Initiation of cancer by CSCs is prominent due to their stemness properties allowing them to accumulate the underlying carcinogenic and mutagenic inducer including inflammation and oxidative stress. CSC further promotes cancer growth and progression by mutual interaction with the microenvironment and harnesses them to favour their own survival, expansion, resistance properties, promotes angiogenesis and metastatic capability. Therefore, CSCs as potential therapeutic targets will be crucial in developing therapies that control cancer and to achieve more improved clinical responses in patients. Unlocking the biology of CSCs in the tumorigenesis and metastasis is key in the development of novel therapeutics for total elimination of CSCs thus improving the treatment modalities.

## Abbreviations

ALDH: Aldehyde dehydrogenase
BMDCs: Bone marrow derived hematopoietic progenitor cells
CAFs: Cancer-associated fibroblasts
COX2: Cyclooxygenase-2
CSCs: Cancer stem cells
DCN: Decorin
DTC: Disseminated tumour cells
ECM: Extracellular matrix
EMT: Epithelial-mesenchymal transition
EPC: Endothelial progenitor cells
EV: Extracellular vehicles
FOXP2: Forkhead box protein P2
HDAC: Histone deacetylase
HGF: Hepatocyte growth factor
LGR5: Leucine-rich repeat-containing G protein-coupled receptor 5
LOX: Lysyl oxidase
LPPs: Lipid peroxidation products
LUM: Lumican
M-CSFs: Macrophage colony stimulating factor
MET: Mesenchymal to epithelial transition
MIFs: Migratory inhibitory factors
MMP: Matrix metalloproteinase
MSC: Mesenchymal stem cells
MSC-EVs: MSC-derived EVs
MSI1: Musashi homolog 1
NK cells: Natural killer cells
OPN: Osteopontin
POSTN: Periostin
RNS: Reactive nitrogen species
ROS: Reactive oxygen species
SDF-1: Stromal derived factors 1
SH: Schistosomahaematobium
SLRPs: Ssmall leucine-rich proteoglycans
SP: Side populations
TACs: Tumour associated cells
TAMs: Tumour-associate macrophage
TANs: Tumour-associate neutrophils
TNC: Tenascins C
